# Patient Outcomes Related to In-Hospital Delays in Appendicectomy for Appendicitis: A Retrospective Study

**DOI:** 10.7759/cureus.23034

**Published:** 2022-03-10

**Authors:** Oliver Claydon, Billy Down, Sidharth Kumar

**Affiliations:** 1 General Surgery, Norfolk and Norwich University Hospitals NHS Foundation Trust, Norwich, GBR; 2 General Surgery, Milton Keynes University Hospital NHS Foundation Trust, Milton Keynes, GBR; 3 General and Colorectal Surgery, Shrewsbury and Telford Hospital NHS Trust, Shrewsbury, GBR

**Keywords:** treatment outcome, time factors, patient discharge, appendicitis, appendicectomy

## Abstract

Background and objective

In many hospitals, the availability of operating theatres and access to senior surgical and anaesthetic support diminish during night hours. Therefore, urgent surgery is sometimes postponed until the following morning rather than performed overnight, if it is judged to be safe. In this study, we aimed to determine if a delay in laparoscopic appendicectomy in cases of acute appendicitis of over 12 hours, analogous to an overnight delay, correlated with worse patient outcomes. Our primary outcome was delayed discharge from the hospital. Our secondary outcomes were appendicitis severity, conversions, and postoperative complications.

Methods

We undertook a retrospective review of the medical records of patients who underwent laparoscopic appendicectomy for appendicitis at a UK district general hospital between 01/01/2018 and 30/08/2019. For each patient, clinical and demographic information, and time of hospital admission, surgery, and discharge were collected. Delayed discharge was defined as "time to discharge" >24 hours after surgery.

Results

A total of 446 patients were included in the study. In 137 patients (30.7%), "time to surgery" was under 12 hours; in 309 patients (69.3%) "time to surgery" was over 12 hours. Of note, 319 patients (71.5%) had a delayed discharge; 303 patients (67.9%) had complicated appendicitis, and 143 patients had severe appendicitis (32.1%). No statistically significant association between "time to surgery" and delayed discharge, appendicitis severity, conversion, or 30-day re-presentations was observed.

Conclusion

Time from admission to the start of appendicectomy did not affect patient outcomes. Short in-hospital delays in appendicectomy, such as an overnight delay, may be safe in certain patients and should be determined based on clinical judgement.

## Introduction

Appendicitis remains one of the most frequent causes of acute surgical hospital admission worldwide [1] and has a lifetime incidence rate of 7-10% [2]. The classical description of the disease’s natural history includes intra-luminal obstruction of the appendix, leading to bacterial overgrowth, transmural inflammation, and eventual perforation. The presumed time-dependent progression of the disease justifies appendicectomy as an emergency procedure [3], which is standard practice in the UK.

Despite the disease’s ubiquity, there is still uncertainty regarding the optimal timing of appendicectomy. Recent literature suggests that active observation, especially in cases of diagnostic doubt, does not increase mortality [4]; this gives credence to the strategy of treatment with antibiotics in the first instance until appendicectomy can be carried out at a safe and convenient time [5,6]. It is conceivable that appendicectomy need not necessarily have the urgency once assigned to it.

The timing of appendicectomy is often determined by both "patient-related" factors (e.g., radiological and biochemical severity of appendicitis, clinical stability, age, comorbidities) and "hospital-related" factors (e.g., time of the day, availability of staff, availability of theatre space). In many institutions, the number of fully-staffed theatres and the immediate availability of senior surgical and anaesthetic support are reduced overnight. Moreover, many patients presenting with appendicitis are judged to be at minimal immediate risk of deterioration. Accordingly, for patients who are clinically stable and with radiologically uncomplicated pathology, the risk of harm arising from delayed access in senior support if problems are encountered intra/postoperatively is often judged to be greater than the risk of patient harm due to sepsis from delayed appendicectomy. In these cases, surgery is often postponed overnight until normal working hours resume, and the patient is given antibiotics, intravenous fluids, and analgesia in the interim [1,6].

The objective of this study was to determine if a short delay in appendicectomy, analogous to an overnight postponement, represents safe management of acute appendicitis. We investigated the association between time elapsed from admission to start of operation ("time to surgery") and patient outcomes. The primary outcome was the time elapsed from the start of the operation to discharge from the hospital ("time to discharge"). Hospital length of stay (HLOS) is one indicator of postoperative recovery [7] and a longer stay is associated with increased cost to the healthcare system [8]. Secondary outcomes were severity of appendicitis, conversion to open appendicectomy, and unplanned 30-day re-presentation for surgical complications.

## Materials and methods

The study was conducted at a UK district general hospital with approximately 550 beds. Patients who underwent appendicectomy for confirmed appendicitis as the primary pathology at our institution between 01/01/2018 and 30/08/2019 were retrospectively enrolled. Exclusion criteria were negative appendicectomies; appendicitis not being the primary pathology treated; operation note not documented; and timings of admission, operation, and discharge not documented. Patient records were reviewed to obtain data on patient demographics, indications for appendectomy, intraoperative findings, procedure details, time of admission, time of the start of the operation, time of discharge, and unplanned hospital re-presentations in the 30-day postoperative period.

All patients either self-presented to our emergency department or were referred from their general practitioner with symptoms of appendicitis. Patients were initially evaluated either by a surgeon or by an emergency department doctor who would refer to the surgeons when the workup indicated suspicion of appendicitis. Patients were admitted immediately after a clinical or radiological diagnosis of appendicitis was made. The decision for and timing of appendicectomy was made by the surgeon, with consideration for both logistical feasibility and clinical urgency. Hospital records were accessed to obtain the time of admission, the time of the start of the operation, and the time of discharge. The "time to surgery" and "time to discharge" were calculated.

Operation notes were reviewed to determine operative details and findings. Based on the documented description of the appearance of the exposed appendix, patients were subclassified into two groups: simple appendicitis and complicated appendicitis. The "complicated appendicitis" group was defined as involving inflamed appendices that were gangrenous, perforated, with necrosis, or associated with an abscess. Uncomplicated descriptions of the inflamed appendix were classified as "simple appendicitis". Patient records were examined to determine if the patient re-presented to the hospital in the 30-day postoperative period. Re-presentations due to a pre-existing issue or planned follow-up were excluded.

For data analysis, we divided patients into groups based on "time to surgery" <12 hours and >12 hours, as a delay of >12 hours is comparable to an appendicectomy being postponed overnight and performed the following day. Many post-appendicectomy patients are well enough to leave the hospital on the day of the operation [9], and same-day discharge is common practice at our institution. For this study, we defined "delayed discharge" as "time to discharge" >24 hours.

Laparoscopic appendicectomy is employed as the standard treatment for appendicitis at our hospital. All patients were treated with intravenous antibiotics (co-amoxiclav; or ciprofloxacin and metronidazole if patients were penicillin-allergic) in the preoperative period. The operations were performed by surgeons experienced in laparoscopic appendicectomy. The decision to convert to open appendicectomy was made at the discretion of the operating surgeon. In cases of complicated appendicitis, postoperative antibiotics were given for at least three days.

Chi-squared tests and Fisher’s exact tests were used to analyse the categorical data. All data analyses were performed using GraphPad Prism version 8.4.3 for macOS (GraphPad Software, San Diego, CA). A p-value <0.05 was considered statistically significant. As this study involved the analysis of pre-recorded anonymised data, ethical approval was not required from the Institutional Review Board of our institution.

## Results

A total of 532 appendicectomies were performed during the period from 01/01/2018 to 30/08/2019. Of these, appendicitis was not present in 60 cases; appendicitis was not the primary pathology being treated in five cases; an operation note was not documented in 10; and timings of admission, operation, or discharge were not recorded in 11. These patients were excluded, leaving 446 patients for the final analysis.

The mean age of the patients was 32 years, and 11.9% of patients were over 55 years in age. Males accounted for 53.4% of patients. At the operation, 303 patients (67.9%) had simple appendicitis and 143 patients (32.1%) had complicated appendicitis. "Time to surgery" was <6 hours in 33 cases (7.40%), 6-12 hours in 104 cases (23.3%), 12-18 hours in 91 cases (20.4%), 18-24 hours in 100 cases (22.4%), and >24 hours in 118 cases (26.5%). Postoperative time was >24 hours in a majority of the patients (n=319, 71.5%).

Of note, 34 patients (7.17%) had an unplanned re-presentation within 30 days of discharge, 22 of which resulted in readmission. The most common re-presentation diagnoses were postoperative pain (n=10) and wound infection (n=8) (Table 1).

**Table 1:** Breakdown of appendicectomy data

		N	%
Total		446	
Time to surgery	0-6 hours	33	7.40
	6-12 hours	104	23.3
	12-18 hours	91	20.4
	18-24 hours	100	22.4
	>24 hours	118	26.5
Time to discharge	0-12 hours	33	7.40
	12-24 hours	94	21.1
	24-36 hours	162	36.3
	36-48 hours	23	5.16
	48-60 hours	46	10.3
	>60 hours	88	19.7
Operative findings	Simple appendicitis	303	67.9
	Complicated appendicitis	143	32.1
Conversions		19	4.26
30-day unplanned re-presentations	Total	34	7.17
	Pain	10	
	Wound issues/infection	8	
	Abdominal collection	5	
	Residual inflammation	4	
	Ileus	2	
	Bleeding per rectum	1	
	Bowel obstruction	1	
	Diarrhoea	1	
	Ileitis	1	
	Reduced mobility	1	
30-day unplanned readmissions		22	4.93

No significant association was demonstrated between "time to surgery" >12 hours and delayed discharge ("time to discharge" >24 hours). Furthermore, we found no significant association between "time to surgery" >12 hours and either complicated appendicitis, conversion, or 30-day re-presentation (Table 2).

**Table 2:** Association between "time to surgery" and outcomes

		Time to surgery <12 hours		Time to surgery >12 hours		P-value	Odds ratio
		N	%	N	%		
Total		137		309			
Time to discharge	<24 hours	38	27.7	89	29.9	0.823	1.05
	>24 hours	99	72.3	220	70.1		
Operative findings	Simple appendicitis	90	65.7	213	69.4	0.498	1.16
	Complicated appendicitis	47	34.3	96	30.6		
30-day re-presentation		9	6.57	25	6.64	0.578	1.25
Conversions		9	6.57	10	3.24	0.108	0.476

Delayed discharge was significantly associated with patient age and complicated appendicitis. Patients whose discharges were delayed >24 hours were more than three times likely to be over 55 years (p=0.00399) and were more likely to have had complicated appendicitis (p<0.0001) than those discharged earlier (Table 3).

**Table 3:** Predictors of "time to discharge" *Indicates statistically significant result at p<0.05 significance level

		Time to discharge <24 hours		Time to discharge >24 hours		P-value	Odds ratio
		N	%	N	%		
Total		127		319			
Age >55 years		6	4.72	46	14.4	0.00399*	3.40
Males		72	56.7	166	52.0	0.374	0.829
Operative findings	Simple appendicitis	115	90.6	174	54.0	<0.0001*	0.125
	Complicated appendicitis	12	9.45	145	45.0		
Conversions		4	3.15	15	4.70	0.462	1.52

Of note, 30-day re-presentations were positively associated with male sex, delayed discharge, conversions, and complicated appendicitis. Patients who had a re-presentation were more than three times likely to have had a delayed discharge (Table 4).

**Table 4:** Predictors of unplanned 30-day re-presentations *Indicates statistically significant result at p <0.05 significance level

		Re-presentation		No re-presentation		P-value	Odds Ratio
		N	%	N	%		
Total		34		412			
Age ≥55 years		7	20.6	46	11.2	0.103	0.485
Males		25	73.5	213	51.7	0.0142*	0.385
Time to surgery	<12 hours	9	26.5	128	31.1	0.578	1.25
	>12 hours	25	73.5	284	68.9		
Time to discharge	<24 hours	4	11.8	123	29.9	0.0246*	3.19
	>24 hours	30	88.2	289	70.1		
Operative findings	Simple appendicitis	17	50.0	286	69.4	0.0197*	2.27
	Complicated appendicitis	17	50.0	126	30.6		
Conversion		6	17.6	13	3.16	<0.0001*	0.152

## Discussion

In recent years, a debate about the urgency and timing of appendicectomy in cases of acute appendicitis has emerged. Previously, appendicitis was regarded as a surgical emergency necessitating appendicectomy as soon as possible [10]. Recent research has challenged the assumption that any delay in surgery is unsafe, and many institutions, recognising the practical difficulties often encountered in operating overnight, treat appendicitis with antibiotics until the operation can be performed safely at an appropriate time.

Studies confirming the time-dependent nature of appendicitis severity have been published. A significant delay from symptom onset to surgery appears to be associated with complications and poor outcomes, as expected. Ditillo et al. [3], in a retrospective study of over 1000 patients, found that both appendicitis severity and postoperative complication rate increase with greater time from symptom onset to operation, with a delay over 71 hours being associated with a 13-fold increase in the risk of advanced appendicitis. These results were mirrored in Saar et al.’s [11] prospective study of 266 patients, in which they reported a stepwise increase in the risk of postoperative complications with delays from symptom onset to operation, and complicated appendicitis (perforation, regional abscess, diffuse peritonitis) associated with a delay of over 36 hours.

A shorter delay from hospital admission to appendicectomy appears not to significantly affect patient outcomes; however, Kim et al.’s [2] retrospective study of over 4000 patients found no significant effect of the time delay from hospital admission to operation on perforation rates or postoperative complications. Surana et al.'s [12] retrospective study of 695 paediatric patients also failed to show a significant difference in perforation rate between patients who had an appendicectomy within six hours of admission and those waiting 6-18 hours for the procedure.

Similarly, a large meta-analysis [13] of 8858 patients in 11 studies demonstrated that a delay of 12-24 hours after admission did not increase the risk of complex appendicitis (although it did demonstrate separately that the risk of surgical site infection and 30-day readmission increased after a 48-hour delay). It is notable that these studies investigated in-hospital delay rather than total time from symptom onset to operation. The apparent lack of disease progression during the interval period demonstrates the importance of perioperative medical treatment (antibiotics and intravenous fluids) in cases of a relatively brief preoperative delay.

Some controversy exists in the literature regarding the association between "time to surgery" and total HLOS. Some authors have described such an association. For example, Eko et al.’s [10] retrospective review of 396 patients demonstrated a significantly shorter HLOS if "time to surgery" is <18 hours, although "time to surgery" did not affect perforations or postoperative complication rates. Udgiri et al. [14] demonstrated similar findings, with "time to surgery" >10 hours being associated with greater HLOS. In contrast, Lee et al. [15] found no such association in a larger review of 1076 patients, and additionally no association with advanced pathology or postoperative complications. These findings have been replicated in a study by Yardeni et al. [5] looking solely at paediatric appendicectomies, with no difference demonstrated in HLOS, perforation rates, or complication rates between patients waiting less than six hours and those waiting more than six hours. Furthermore, a systematic review [16] including 34 papers found no association between a short "time to surgery" (<24 hours) and HLOS.

Our retrospective study of 446 patients found no association between "time to surgery" and delayed discharge. Also, we found no association between "time to surgery" and complicated appendicitis, conversions, or unplanned 30-day re-presentations. We, therefore, conclude that a short delay in appendicectomy, e.g., the operation being postponed overnight, can be a part of the safe management of acute appendicitis. We also found delayed discharge >24 hours to be strongly associated with patient age and severity of appendicitis. Additionally, 30-day re-presentations were associated with delayed discharge, complicated appendicitis, and most strongly with conversions.

There are some limitations to our study, including the relatively small sample size and the fact that a single-centre study may not be representative of the characteristics of the broader population or common management procedures in other institutions. More importantly, we acknowledge that our study did not take into account the preoperative clinical rationale regarding the timing of appendicectomy in each case. In many patients who underwent appendicectomy overnight, high clinical severity (e.g., systemic sepsis, uncontrolled pain) may have justified an out-of-hours operation. Therefore, our findings do not recommend delaying appendicectomies overnight across the board, but rather advocate the use of clinical judgement.

## Conclusions

Based on our findings, delay from admission to laparoscopic appendicectomy did not affect the time to discharge, appendicitis severity, conversions, or re-presentations. In stable patients with uncomplicated appendicitis, a short in-hospital delay in surgery, e.g., an overnight delay, can be safe. Delayed discharge and 30-day re-presentations were more likely to occur after appendicectomy for complicated appendicitis, and 30-day re-presentations were more likely after conversions.
